# Partisan animosity through the lens of blame: Partisan animosity can be reduced by a historicist thinking intervention

**DOI:** 10.1371/journal.pone.0295513

**Published:** 2024-01-10

**Authors:** Raihan Alam, Michael J. Gill

**Affiliations:** Department of Psychology, Lehigh University, Bethlehem, PA, United States of America; National Taiwan University, TAIWAN

## Abstract

Partisan animosity has been on the rise in America. Partisan animosity involves blame, wherein political partisans blame outparty members for their beliefs and actions. Here, we examine whether a historicist thinking intervention—drawn from research on blame mitigation—can reduce partisan animosity. The intervention consisted of three components: (1) a narrative about the idiosyncratic development of one political opponent paired with (2) a message about how unique life experiences shape *everyone’s* political beliefs and (3) a suggestion that outparty members can be changed by future formative experiences. Experiments 1 and 2 showed that the intervention reduced cold feelings—measured via Feeling Thermometer—towards the outparty for both Democrats and Republicans. Experiments 3 and 4 focused on more specific emotional changes. Experiment 3 showed that, for Democrats, the intervention increased compassion. Experiment 4 showed that, for Republicans, the intervention reduced disgust, disapproval, anger, and contempt, but had no impact on compassion. For Democrats, but not for Republicans, reductions in animosity were mediated by reduced perceptions of control of self-formation, the mediator identified in prior work on historicist thinking and blame mitigation.

## Introduction

Partisan animosity—hostile thoughts, feelings, or behaviors directed towards a political outgroup [1]—has been on the rise in the United States. Indeed, since the 1980s, neutral feelings towards the opposing political party have steadily soured and been replaced by feelings of coldness and hostility [2]. In the past decade, the strength of animus towards the political outparty has eclipsed the strength of warm feelings towards the inparty [2]. Although partisan animosity is not uniquely American, outparty hate is stronger in the United States than in other Western democracies [3]. Such strong partisan animosity has been linked to support for political and social exclusion of the outparty and to endorsement of violence against the outparty [4, 5]. It has also been associated with a tendency to trust the government only when one’s own party is in power and a reduced willingness to compromise with those who have a different viewpoint [6, 7]. With the rise of partisan animosity and an increased understanding of its destructive impact on democracy, social scientists have started examining interventions to reduce it [1, 8–18]. We join with these other scholars in an attempt to tackle the problem of partisan animosity. In two of the studies below, the research procedures are based on those dictated by the rules that Voelkel et al. [17] used to conduct a “tournament” that pitted various interventions to reduce partisan animosity against one another. In two additional studies, we utilize different dependent variables than those pre-registered by Voelkel et al. [17] to provide additional information about the effects of our intervention.

Our approach to the problem of partisan animosity has roots in the psychology of blame.

Indeed, partisan animosity is suffused with blame, with each side viewing the other not merely as “holding different beliefs from mine” but rather as holding beliefs that are unethical, morally repugnant, inhumane, disgusting, and so on. Thus, interventions known to mitigate blame should be relevant for mitigating partisan animosity. Accordingly, we will examine whether a historicist thinking intervention—emphasizing how outparty members’ beliefs were created via a powerful set of formative experiences—can reduce partisan animosity. In a variety of domains (discussed below), historicist thinking has been shown to reduce hostile blame responses, mediated by a reduced perception that the target of blame self-created her morally offensive attributes. In every study below, then, we will test whether a historicist thinking intervention reduces partisan animosity and whether this effect is mediated in the same way as historicist thinking effects in other domains. Before elaborating on our approach, we will review existing work on how to reduce partisan animosity.

## Prior research on how to reduce partisan animosity

One mechanism for reducing partisan animosity involves correcting inaccurate metaperceptions, or mistaken beliefs about what the outparty thinks of one’s own party. Indeed, Democrats and Republicans overestimate the extent to which members of the outparty dehumanize and have prejudice towards them [19], as well as how willing outparty members are to use violence for political goals [13]. Interventions that correct these faulty metaperceptions by presenting accurate data regarding outparty beliefs reduce both the extent to which Democrats and Republicans dehumanize the outparty and support partisan violence [11, 13]. Beyond metaperceptions, people also have highly inaccurate beliefs about the composition of the outparty [8]. For example, in one survey, people guessed that 32% of Democrats are gay, lesbian, or bisexual (actually 6%) and that 38% of Republicans make over $250,000 annually (actually 2.2%). Ahler & Sood [8] showed that correcting such misperceptions reduces partisan hostility.

Whereas work in the prior paragraph emphasizes correcting inaccurate beliefs, other work focuses on perspective taking or empathy. For example, Santos et al. [15] found that priming partisans with the belief that having empathy for the political outparty increases political persuasiveness reduced their levels of partisan animosity. Stanley et al. [16] found that hostile character derogation of the outparty was reduced when partisans were encouraged to “get inside the heads” of their opponents by considering some compelling arguments, reasons, and evidence for outparty political stances. Relatedly, Kubin et al. [10] found reductions in character derogation of the outparty when partisans read about how political opponents developed their views as a result of personal experiences of harm to self or loved ones (rather than as a result of abstract factual information). Relatedly, Waytz et al. [18] showed that partisan animosity can be reduced if ideological opponents come to see the other side as motivated by love for their ingroup instead of by hatred towards their outgroup (which is how the other side typically sees itself).

Voelkel et al. [17] conducted an intervention tournament that examined 25 interventions aimed at reducing partisan animosity and anti-democratic attitudes, with many of the tested interventions based on the prior research above. They found that interventions that showcased positive outparty members, or a shared cross-partisan identity, were most effective at reducing partisan animosity. For example, the intervention with the largest effect size was one that showed individuals with opposing political views working together and choosing to cordially discuss their differences over a drink. Another effective intervention emphasized how most Americans from both major political parties value democracy, and therefore, share a common American identity of being supporters of democracy.

In short, prior work has shown many conceptual approaches for reducing partisan animosity, such as correcting inaccurate beliefs about the outparty and promoting perspective taking. Correcting inaccurate beliefs reduces outparty dehumanization and support for partisan violence by providing accurate information about outparty beliefs [11, 13], whereas perspective-taking interventions foster a more compassionate understanding of political opponents by encouraging individuals to consider their viewpoints and motivations [10, 15, 16, 18]. We believe that exploring the role of blame in the context of partisan animosity can be useful for deriving new interventions and for understanding why prior interventions are effective. Now, we describe our blame-based theoretical approach to reducing partisan animosity.

## Our approach: Blame and historicist thinking

As mentioned, our approach to the problem of partisan animosity has roots in the psychology of blame. Our conceptual approach to blame follows that developed by Gill and Cerce (see [20] for elaboration) and also draws heavily on work from Malle and his colleagues [21]. Blame begins with the perception that a person or a group has violated a norm or a moral principle. We define *blame* as the hostile affective response—anger, irritation, outrage—triggered by this perception [20, 22]. For example, in the context of ideological animosity, blame might sound like this: *It infuriates me that they take money from hardworking people and give it to lazy bums*! Blame from the other side of the political aisle might sound like this: *It infuriates me that they refuse to share their massive wealth with people in dire need*! Whereas blame *per se* is affectively charged, it grows out of social cognitive processes that analyze various features and capacities of the potentially blameworthy target. Specifically, the intensity of blame—the force of its hostility—depends on several factors such as the perceived intentionality of the norm-violator [21], the specific reasons behind her actions [21], whether his actions were freely chosen [23], and whether her moral character was self-created [23].

Although the blame literature tends to focus on interpersonal interactions, blame can also operate at the group level. For example, people become angry at groups that are perceived to act unjustly [24], they hate groups that engage in violence [4], and they experience hostile emotions toward groups associated with terrorist attacks [25]. We note that our concept of blame has significant overlap with the concepts discussed as drivers of partisan animosity in Finkel et al.’s prominent report, *Political Sectarianism in America* [2]. Finkel and colleagues argue that partisan animosity is driven by the confluence of three main psychological factors: Othering (seeing the outparty as essentially different from the inparty), aversion (strongly disliking and distrusting the outparty), and moralization (viewing the outparty as immoral). Blame can be seen as aversion caused by moralization.

In the studies described below, we draw specifically from the literature on historicist narratives and their role in blame mitigation. *Historicist narratives* are story-like descriptions of how an individual developed her character and worldview through formative life experiences [23]. Historicist narratives have been shown to reduce blame of a target person for a variety of negative actions, including bullying and homicide [23], arrogance [26], laziness [27] and, of most relevance here, nasty communication that derogated the listener’s ideological views [9]. Historicist narratives reduce blame by implying that the offending individual lacked *control of self-formation*, or the capacity to self-determine his own character. Instead, the narrative implies that the character and worldview of the offending person were “implanted” by powerful formative life experiences, such as physical and verbal abuse from parents, indulgent parenting, growing up in a politically homogenous community, strong religious socialization, an absence of positive role models, and so on. This mediating effect of perceived control of self-formation has been replicated in numerous studies [9, 23, 26–31]. Prior work has also ruled out the possibility that the blame-mitigating effects of historicist narratives merely represent “individuation” effects (i.e., changes sparked simply by learning more about the targets’ individual attributes) [9, 23]. For example, Gill and Cerce [23] showed that individuating information that did not explain character development (i.e., was not a historicist narrative) had a non-significant effect on blame mitigation. Furthermore, Gill, Alam, and Nagelhout [9] showed that historicist narratives reduced expressions of partisan hostility compared to a control condition in which individuating information was presented.

As mentioned, one recent study showed that historicist narratives can reduce animosity toward an individual outparty member [9]. In that work, all participants read a “tweet” from an outparty member that was harshly critical of the participant’s ideological position. Participants were given an opportunity to “tweet” back at her. Gill et al. [9] varied what type of background information participants had about the nasty tweeter prior to replying to her. Some participants learned only bland, non-diagnostic information about her (e.g., she is from Denver, she doesn’t like the cold winters), whereas others learned how her life history—experiences in her family, in church, and so on—shaped her political beliefs. Results showed that the historicist narrative reduced the harshness of participants’ tweeted replies to their critic, mediated by a reduction in perceived control of self-formation. Gill et al. [9] presented follow-up studies in which, rather than receiving a specific historicist narrative about the nasty tweeter, participants instead read a general message about how life experiences shape *everyone’s* political beliefs. They found that, at least for liberals, this *general historicist reminder* reduced how harshly they responded to the nasty tweeter (mediated, again, by reduction in perceived control of self-formation).

Unlike Gill et al. [9], here we are concerned with changing attitudes toward the outparty *as a whole* rather than communication with a particular outparty member. Our intervention will draw on the materials from all of the Gill et al. [9] studies. Although the bulk of our intervention will focus on historicist thinking per se, one portion of the intervention focuses on the perceived malleability—the potential for belief change—of the outparty. We view perceived malleability as an implication of historicist thinking: If she was shaped by formative experiences earlier in life (historicist thinking), then she can be shaped by them again (future malleability [32]).

## The present experiments

### Overview of experiments

Below, we present 4 experiments, using a between-subjects design for each (historicist thinking intervention vs control). The first two stick closely to the study procedure protocols developed by Voelkel et al. [17] for their intervention tournament. The first one focuses on self-identified Democrats and the second focuses on self-identified Republicans. Both experiments examine whether a historicist thinking intervention can reduce partisan animosity. The intervention includes a concrete historicist narrative regarding a particular outparty member (as in Gill et al. [9]), a follow-up “generalizing statement” which suggests that *every* outparty member has a unique story about formative influences on his or her beliefs (similar to Gill et al. [9]), and, finally, an “implications statement" suggesting that outparty members can be changed by future formative experiences in the same way that they have been affected by past formative experiences.

In the first two experiments, our measure of partisan animosity was the feeling thermometer, which was one of the two measures dictated by the Voelkel et al. [17] tournament protocols. Their other measure was generosity in a dictator game played with a member of the political outparty. We focus in the studies below on the feeling thermometer because pilot testing showed that our intervention affected feelings toward the outparty more consistently than generosity toward them. In addition to being a preregistered and required measure per Voelkel et al. [17], the feeling thermometer is a primary method for measuring affective polarization in the broader literature on the topic [1, 33–38].

In all studies below, we will use one-tailed tests of statistical significance. This is justified on four grounds: (1) One-tailed tests were used by Voelkel et al. [17] in their partisan animosity intervention tournament, and we want our results to be directly comparable to theirs, (2) The impact of historicist narratives on blame mitigation is well-established and there is not a single study in which historicist narratives have increased blame [9, 23, 26–31], and (3) Interventions to reduce partisan animosity generally have small effects (e.g., in Voelkel et al. [17], average |*d*| = .25 for the 23 interventions that had a significant effect, with roughly 40% having effect sizes of d < .20); thus, the risk of Type II Error is high (and a one-tailed test lowers that risk). To avoid a threat to internal validity, we decided to not remove participants that failed our manipulation or attention checks because these checks were presented post-treatment. The exclusion of participants after exposure to an experimental condition can lead to post-treatment bias [39]. All analyses were conducted in SPSS version 28. For all studies, we did not have access to information that could identify individual participants during or after data collection.

## Experiment 1: Can we reduce Democrats’ animosity—assessed via feeling thermometer—toward Republicans?

Experiment 1 focused on Democrats. Participants either received our historicist thinking intervention or not. Our key prediction was that our intervention would reduce the amount of partisan animosity Democrats feel towards Republicans, mediated by reduced belief in control of self-formation.

### Method

#### Ethics statement

Experiment 1 and all subsequent experiments below were approved by Lehigh University’s Institutional Review Board (Proposal ID: 1711796–2). Participants provided informed consent by clicking an "agree" button after reading the IRB-approved consent form. All data and study materials can be found at: https://osf.io/t24w8/.

#### Participants

We used Prolific’s prescreening feature to recruit 703 participants who self-categorized as Democrats. These participants are a combined sample of different Democrats who took the survey in either May (*N* = 303) or September of 2022 (*N* = 400). Six-hundred ninety-nine participants completed all relevant portions of the survey (i.e., control of self-formation and partisan animosity items). For all studies, information about precisely which participant responses are missing can be found in the S1 File, under the *Missing Data* section. We found a marginal difference in partisan animosity between the two samples *t*(699) = -1.92, *p* = .06 (two-tailed; *d* = .15; 95% *CI*: -.30, .003). We will control for time of data collection in an analysis below to ensure that it does not modify our results. Also, because we peeked at the data before collecting the second round—our effect was statistically significant but small when we peeked—we adjusted our critical *p*-value accordingly [40]. Our new critical *p*-value, calculated using Sagarin et al.’s *pcrit* function in *R*, is *p* = .04 (one-tailed). Based on a sensitivity analysis computed via G*Power [41], this sample size provided us with 80% power to detect a small effect size (*d* = .20; *t*-test of independent means, α = .04, one-tailed).

#### Procedure

After providing informed consent, participants learned that they would fill out ratings related to politics. They were randomly assigned to a *control condition* (*N* = 351) or to the *historicist thinking intervention (N* = 352). In the control condition, participants went straight to our measure of partisan animosity (described below).

In the historicist intervention condition, we first introduced participants to a Republican named Sabrina, showing them a photo of her (available in S1 File). Next, they read a paragraph describing how Sabrina developed her conservative worldview (e.g., *Sabrina is 19 years old*. *She grew up in an Evangelical Christian home…*; full text can be seen in S1 File). Then, they read our generalizing statement:

*Although Sabrina’s story is not the same as the story for all Republicans*, *it is nevertheless true that every person has a story behind his or her political views*. *Indeed*, *every individual’s beliefs and attitudes are created in the context of his or her family*, *personal upbringing*, *geography*, *media exposure*, *spiritual and religious background*, *educational background*, *class*, *etc*. *No one becomes who they are all by themselves*, *but rather each person is forged by their surroundings and life experience*s.

Finally, the intervention included a statement that connected historicism to malleability:

*Just as every person’s belief system is formed through a variety of formative experiences*, *belief systems can also change via formative experiences*. *These formative experiences can include new learning*, *conversations with those who see things differently*, *moving to a new part of the country*, *etc*.

Following exposure to our intervention, participants in the intervention condition completed our measure of partisan animosity, the feeling thermometer. This measure required our Democrat participants to rate their feelings toward Republicans on a scale ranging from 0 (very cold) to 100 (very warm). Because our focus is on animosity, we reverse coded the feeling thermometer such that high scores indicated “very cold” feelings. Overall, our Democrat participants had very negative feelings toward Republicans (*M* = 80, *SD* = 18).

After participants completed the dependent measures, they completed demographic items and were then fully debriefed and thanked for their participation.

### Results

Did our historicist thinking intervention reduce partisan animosity? We computed a two-sample *t*-test (control vs. intervention) to test this. The analysis revealed a significant effect of our intervention, *t*(699) = 2.57, *p* = .005 (*d* = .19; 95% *CI*: .05, .34). This reflected the fact animosity toward Republicans was reduced by our intervention (*M =* 79) as compared to the control condition (*M* = 82). Because of the marginal difference in animosity between Time 1 and Time 2 data collection, we conducted a follow-up 2(condition: control, historicist thinking intervention) X 2(time of data collection: Time 1, Time 2) ANOVA to examine whether our results were impacted by time of data collection. This analysis replicated the effect of condition, *F*(1, 697) = 7.00, *p* = .008, and the marginal effect of time, *F*(1, 697) = 3.81, *p* = .051. A non-significant interaction indicated that time of data collection did not moderate the impact of our intervention, *F*(1, 697) = .33, *p* = .57. The results also withstood an analysis controlling for pre-treatment collected demographic variables of age, sex, race, and employment status. See the *Analyses Not Presented in the Main Text* section of the S1 File (for this study and for all studies below any significant effects of the control variables are described there).

**Fig 1:**
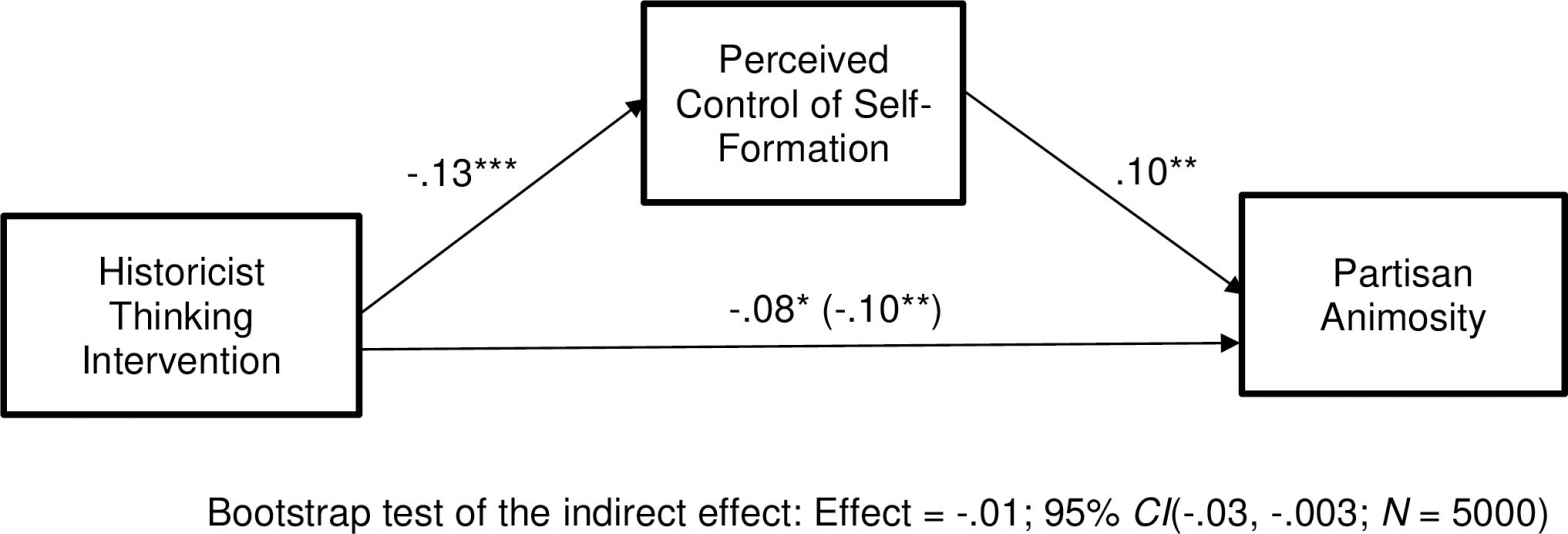
Experiment 1: Among Democrats, the historicist thinking intervention lowers perceived control of self-formation, thereby reducing partisan animosity. Coefficients are standardized. *p < .05, **p < .01, ***p < .001.

Next, we computed our mediation model. We used Model 4 from Hayes’ (2018) PROCESS software. There is reasonable skepticism of mediation analyses, and we encourage readers to view such correlational models with appropriate caution (See S1 File for information regarding drawbacks of correlation-based mediation models under the heading *Drawbacks of Correlational Tests of Mediation*). All variables were standardized prior to computing the model, so the presented coefficients are standardized beta weights. Results can be seen in Fig 1. As can be seen there, the historicist intervention significantly reduced perceived control of self-formation, *t*(697) = -3.51, p < .001, and perceived control of self-formation was positively related to partisan animosity, *t*(696) = 2.65, *p* = .004. This was a significant indirect effect, as can be seen by looking at the bootstrap test results beneath the model. The direct effect of our intervention remained significant even after controlling for the mediator, *t*(696) = -2.19, *p* = .01 suggesting either that there are other mediators of the effect or that our measure of control of self-formation is imperfect.

## Experiment 2: Can we reduce Republicans’ animosity—assessed via feeling thermometer—toward Democrats?

Experiment 2 focused on Republicans. The procedure and predictions were identical to Experiment 1.

### Method

#### Participants

We used Prolific’s prescreening feature to recruit 650 participants who self-categorized as Republicans. These participants are a combined sample of different Republicans who took the survey in either May (*N* = 300) or October/November of 2022 (*N* = 350). Our peeking-adjusted critical *p*-value, calculated as in Experiment 1, is *p* = .04.

After combining the two datasets, we filtered out cases with the same Prolific ID, only keeping the first case assigned to a condition (*N* = 2; both cases were assigned to the intervention condition). Six-hundred forty-eight participants completed all relevant portions of the survey. We found no difference in partisan animosity between the two samples *t*(644) = -1.59, *p* = .11 (*d* = .13; 95% *CI*: -.28, .03). Based on a sensitivity analysis computed via G*Power [41] this sample size provided us with 80% power to detect a small effect size (*d* = .20; *t*-test of independent means, α = .04, one-tailed).

#### Procedure

The procedure was identical to Experiment 1. Participants were randomly assigned to a *control condition* (*N* = 326) or to the *historicist thinking intervention* (*N* = 322). Participants in the control condition went straight to the dependent variables. Participants in the intervention condition saw the photo of “*Democrat* Sabrina” and read a paragraph that described how Sabrina developed her *liberal* worldview (e.g., *Sabrina is 19 years old…grew up in a liberal bubble…*; full text available in S1 File). They then read the statement that generalized from Sabrina to Democrats as a whole. Finally, as in Experiment 1, participants read the implications statement emphasizing how formative influences can make a person’s beliefs malleable in the future.

Following exposure to our intervention, participants in the intervention condition completed the feeling thermometer, reversed scored as in Experiment 1. Overall, our Republican participants had moderately negative feelings toward Democrats (*M* = 68, *SD* = 22).

After participants completed the dependent measures, they completed demographic items and were then fully debriefed and thanked for their participation.

### Results

Did our historicist thinking intervention reduce partisan animosity? We computed a two-sample *t*-test (control vs. intervention) to test this. The analysis revealed a significant effect of our intervention, *t*(644) = 2.57, *p* = .005 (*d* = .20; 95% *CI*: .05, .36). This reflected the fact animosity toward Democrats was reduced by our intervention (*M =* 65) as compared to the control condition (*M* = 70). Thus, our intervention is successful at reducing partisan animosity for both Democrats and Republicans. The results also withstood an analysis controlling for pre-treatment collected demographic variables of age, sex, race, and employment status (see S1 File).

**Fig 2:**
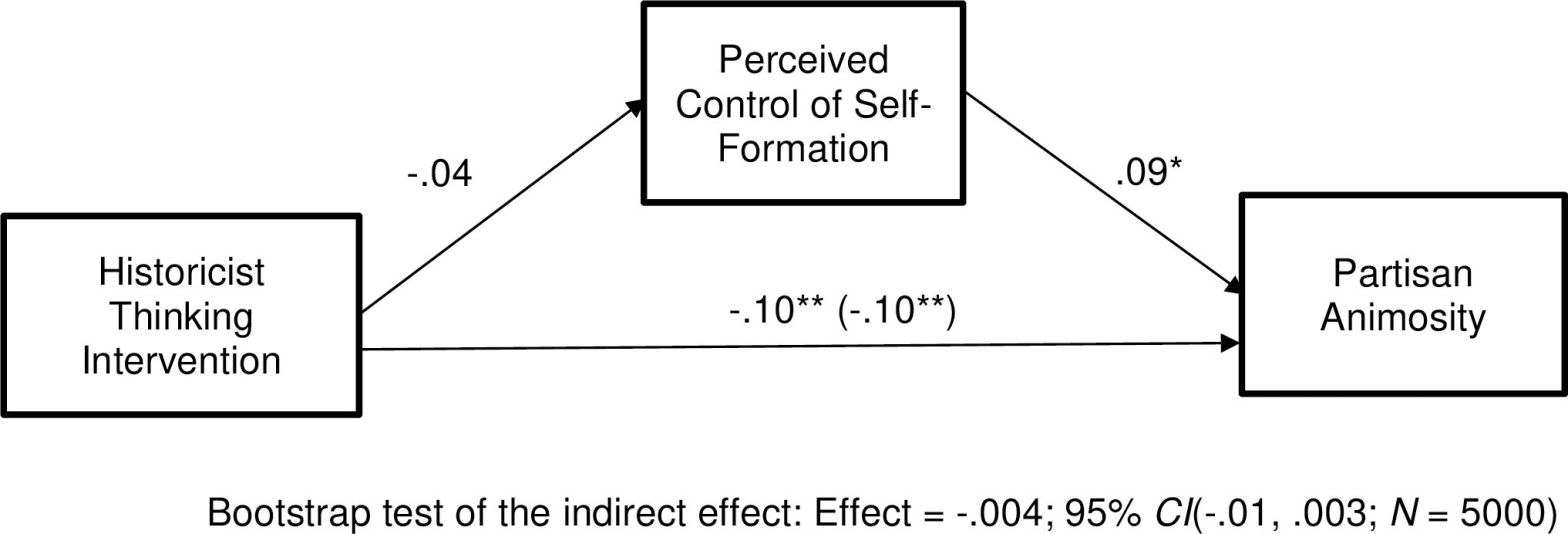
Experiment 2: Among Republicans, the historicist thinking intervention reduces partisan animosity, but not through perceived control of self-formation. Coefficients are standardized. *p < .05, **p < .01, ***p < .001.

Next, we computed our mediation model. We used Model 4 from Hayes’ (2018) PROCESS software. All variables were standardized prior to computing the model, so the presented coefficients are standardized beta weights. Results can be seen in Fig 2. As can be seen there, the historicist intervention did not significantly reduce perceived control of self-formation, *t*(644) = -1.10, *p* = .14, but perceived control of self-formation was positively related to partisan animosity, *t*(642) = 2.23, *p* = .01. This was not a significant indirect effect, as can be seen by looking at the bootstrap test results beneath the model. Crucially, then, although our intervention did succeed in reducing partisan animosity among Republicans, it appears that this did not happen via reductions in control of self-formation.

## Experiment 3: Can we reduce Democrats’ animosity—measured via moral emotions—toward Republicans?

Experiments 3 and 4 replace the feeling thermometer measure of animosity with separate measures of a variety of moral emotions commonly studied in the literature on blame (e.g., outrage, disgust, hate, compassion). This will enable us to achieve a more fine-grained view of precisely what is changed by our intervention. That is, whereas the feeling thermometer assesses animosity using a single dimension ranging from warm to cold, Experiments 3 and 4 will measure animosity via seven distinct moral emotions and identify which particular emotions are affected by our intervention. Are we primarily reducing hate? Increasing compassion? Treating these emotions separately is justified by prior literature regarding their different elicitors and/or different social functions [42–45].

### Method

#### Participants

In October of 2022, we used Prolific’s prescreening feature to recruit 400 participants who self-categorized as Democrats. Three-hundred eighty-nine participants completed all relevant portions of the survey (i.e., control of self-formation and moral emotions ratings). Based on a sensitivity analysis computed via G*Power [41], this sample size provided us with 80% power to detect a small effect size (Pillai’s Trace *V* = .035; one-way MANOVA, α = .05).

#### Procedure

After providing informed consent, participants learned that they would fill out ratings related to politics. They were randomly assigned to a *control condition* (*N* = 202) or to the *historicist thinking intervention (N* = 198). In the control condition, participants went straight to our measures of moral emotions (described below).

The historicist intervention condition was identical to Experiment 1. That is, participants were introduced via photo to a Republican named Sabrina and then read a paragraph describing how Sabrina developed her conservative worldview. Then, they read our generalizing statement (i.e., everyone has a story of belief formation) and the statement connecting historicism to malleability.

Participants in both conditions rated their emotional responses to Republicans on a five-point scale with endpoints labeled *Not at All* (1) and *Strongly* (5). We view all these emotions as indicators of animosity (or its absence). They read a prompt (*Toward Republicans I feel…*) and then rated a series of emotion words. There were two items tapping the positive moral response of *compassion* (*compassion*, *sympathy*; *M* = 1.81, *SD* = .81, *α* = .90). The items also included a variety of negative moral emotional responses that varied in intensity. Low intensity items included a single item tapping *disapproval* (*M* = 4.19, *SD* = 1.03) and two items tapping *disappointment* (*disappointed*, *let down*; *M* = 3.99, *SD* = 1.15, *α* = .92). The more intense moral emotional responses included *anger* (*anger*, *outrage*, *infuriated*; *M* = 3.26, *SD* = 1.22, *α* = .95), *contempt* (*disrespect*, *contempt*; *M* = 2.53, *SD* = .84, *α* = .81), *disgust* (*disgust*, *repulsed*, *sickened*; *M* = 3.09, *SD* = 1.36, *α* = .96), and *hatred* (*hatred*, *hostility*, *loathing*, *scorn*; *M* = 2.73, *SD* = 1.20, *α* = .93). Overall, the means suggest that our Democrat participants had very little compassion for Republicans. In contrast, they reported high levels of disapproval and disappointment. The more intense negative emotions—anger, contempt, disgust, hatred—fell in between these two extremes, receiving ratings close to the scale midpoint. Participants also rated perceived control of self-formation on slightly revised items that referred to “Each individual Democrat” having control of self-formation as opposed to “Democrats as a whole” (*M* = 3.65, *SD* = .88, *α* = .84). We reasoned that participants might find it odd to think of a “group as a whole” having control of self-formation.

After participants completed the dependent measures, they completed demographic items and were then fully debriefed and thanked for their participation.

### Results

Did our historicist thinking intervention reduce partisan animosity? To test this, we computed a one-way MANOVA (control vs. intervention) analyzing all of the moral emotions measured. The analysis revealed a significant effect of our intervention on Democrats’ feelings toward Republicans, *F* (7, 381) = 2.74, *p* = .005; Pillai’s Trace *V* = .05, partial η^2^ = .05, 90% *CI*: .01, .07. The results also withstood an analysis controlling for pre-treatment collected demographic variables of age, sex, race, and employment status (see S1 File).

To determine the particular moral emotions affected by our intervention, we did follow up tests focused on each specific emotion. We corrected for multiple comparisons using the Benjamini-Hochberg procedure [46]. First, we ranked individual p-values in ascending order. Next, we took the product of our false discovery rate (.05 as used by Voelkel and colleagues [17]) and p-value rank and divided it by the number of outcomes we tested (7). Results can be seen in Table 1. As can be seen there, our intervention’s effect on compassion remained significant, while its effects on disgust and anger became marginal. Hence, our historicist thinking intervention significantly increased Democrats’ compassion for Republicans, suggesting that the reduced animosity of Democrats consists primarily of an increase in warm-hearted feelings based on our intervention.

**Table 1:** Experiment 3: Impact of the historicist thinking intervention on moral emotional responses.

	*M*_Control_ (*SE*)	*M*_*Intervention*_ (*SE*)	*f-*value	Partial η^2^	*p-* value	Rank	Critical *p-*value	Comparison
Compassion	1.69 (.06)	1.94 (.06)	9.16	.02	.002	1	.007	Significant
Disgust	3.24 (.10)	2.95 (.10)	4.57	.01	.02	2	.01	Non-significant
Anger	3.39 (.09)	3.15 (.09)	3.91	.01	.025	3	.02	Non-significant
Hatred	2.80 (.09)	2.66 (.09)	1.19	.003	.14	4	.03	Non-significant
Contempt	2.57 (.06)	2.49 (.06)	.84	.002	.18	5	.04	Non-significant
Disapproval	4.24 (.07)	4.15 (.07)	.81	.002	.184	6	.04	Non-significant
Disappointment	3.95 (.08)	4.03 (.08)	.53	.001	.23	7	.05	Non-significant

*Note*. *df* = 388

**Fig 3:**
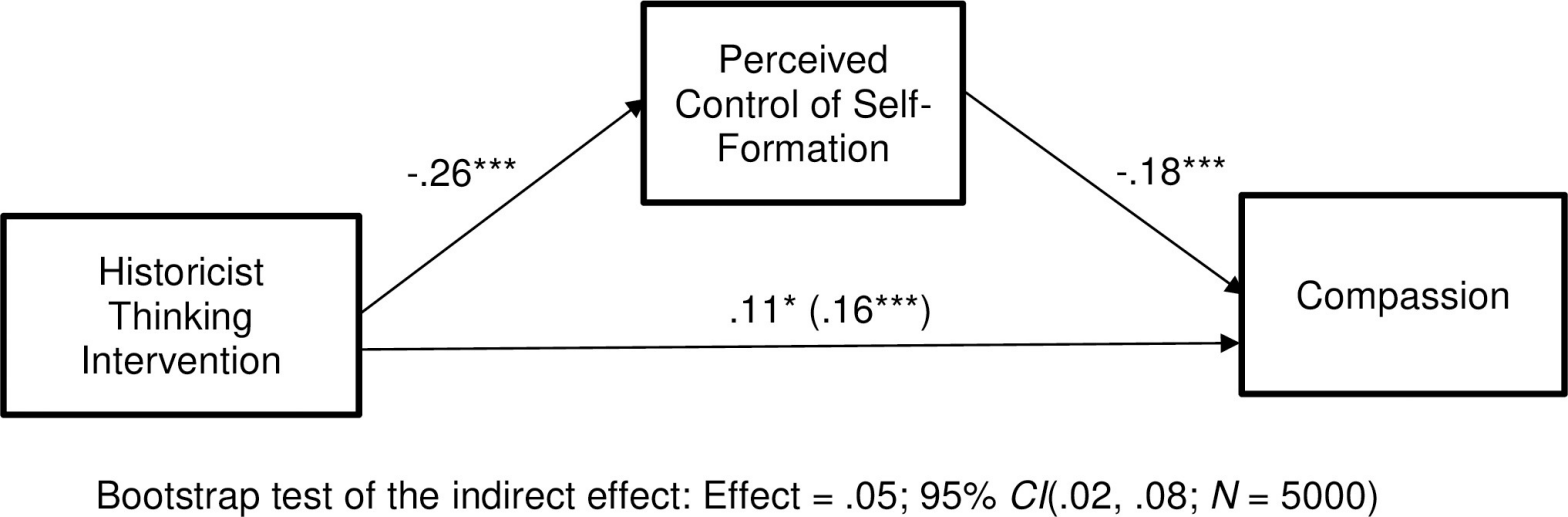
Experiment 3: Among Democrats, the historicist thinking intervention lowers perceived control of self-formation, thereby increasing compassion. Coefficients are standardized. *p < .05, **p < .01, ***p < .001.

Next, we computed our mediation model. We computed it for compassion, the only statistically significant effect discussed in the prior paragraph. We used Model 4 from Hayes’ (2018) PROCESS software. All variables were standardized prior to computing the model, so the presented coefficients are standardized beta weights. Results can be seen in Fig 3. As can be seen there, the historicist intervention significantly reduced perceived control of self-formation: *t*(397) = -5.43, *p* < .001. In turn, perceived control of self-formation was associated with reduced compassion, *t*(396) = -3.48, *p* < .001. This was a significant indirect effect, as can be seen by looking at the bootstrap test results beneath the model. The direct effect of our intervention remained significant even after controlling for the mediator, *t*(396) = 2.25, *p* = .01 suggesting either that there are other mediators of the effect or that our mediator is not well-measured. The significant effects in this model were generally larger than the effects found in Experiment 1. The link between the intervention and perceived control of self-formation was two times larger than in Experiment 1 and the link between the intervention and compassion was 80% larger than its link with partisan animosity in Experiment 1. These larger effects might be due to the more reliable emotion measures and/or to the revised control of self-formation items.

Experiment 3, then, provides a conceptual replication of Experiment 1: Our historicist thinking intervention reduces partisan animosity and—at least for Democrats—this appears to involve increased compassion toward Republicans. This change in emotion was, as in Experiment 1, mediated by reduced perceptions of control of self-formation.

## Experiment 4: Can we reduce Republicans’ animosity—measured via moral emotions—toward Democrats?

Experiment 4 focused on Republicans. The procedure and predictions were identical to Experiment 3.

### Method

#### Participants

In October of 2022, we used Prolific’s prescreening feature to recruit 401 participants who self-categorized as Republicans. Three-hundred ninety-seven participants completed all relevant portions of the survey. Based on a sensitivity analysis computed via G*Power [41], this sample size provided us with 80% power to detect a small effect size (Pillai’s Trace *V* = .035; one-way MANOVA, α = .05).

#### Procedure

After providing informed consent, participants learned that they would fill out ratings related to politics. They were randomly assigned to a *control condition* (*N* = 201) or to the *historicist thinking intervention (N* = 200). In the control condition, participants went straight to our measures of moral emotions (described below).

The historicist intervention condition was identical to Experiment 2. That is, participants were introduced via photo to a Democrat named Sabrina and then read a paragraph describing how Sabrina developed her liberal worldview. Then, they read our generalizing statement (i.e., everyone has a story of belief formation) and the statement connecting historicism to malleability.

Participants in both conditions rated their emotional responses to Democrats on a five-point scale with endpoints labeled *Not at All* (1) and *Strongly* (5). We view all these emotions as indicators of animosity (or its absence). The set of emotions and the items used to measure them were identical to Experiment 3: *Compassion (M* = 2.20, *SD* = 1.01, *α* = .91), *disapproval (M* = 3.74, *SD* = 1.23), *disappointment (M* = 3.61, *SD* = 1.30, *α* = .93), *anger (M* = 2.57, *SD* = 1.18, *α* = .94), *contempt (M* = 2.20, *SD* = .84, *α* = .82), *disgust* (*M* = 2.48, *SD* = 1.34, *α* = .96), and *hatred (M* = 2.07, *SD* = 1.10, *α* = .92). Overall, the means suggest that our Republican participants had very little compassion for Democrats. In contrast, they reported high levels of disapproval and disappointment. The more intense negative emotions—anger, contempt, disgust (but not hatred)—fell in between these two extremes, receiving ratings somewhat below the scale midpoint. Participants also rated perceived control of self-formation using the same revised items as in Experiment 3 (*M* = 3.78, *SD* = .83, *α* = .82).

After participants completed the dependent measures, they completed demographic items and were then fully debriefed and thanked for their participation.

### Results

Did our historicist thinking intervention reduce partisan animosity? To test this, we computed a one-way MANOVA (control vs. intervention) analyzing all of the moral emotions measured. The analysis revealed a significant effect of our intervention on Republicans’ feelings toward Democrats, *F* (7, 389) = 2.18, *p* = .02; Pillai’s Trace *V* = .04, partial η^2^ = .04, 90% *CI*: .001, .06. The results also withstood an analysis controlling for pre-treatment collected demographic variables of age, sex, race, and employment status (see S1 File).

To determine the particular moral emotions affected by our intervention, we did follow up tests focused on each specific emotion. We corrected for multiple comparisons using the Benjamini-Hochberg procedure [46] as we did for Experiment 3. Results can be seen in Table 2. As can be seen there, our intervention’s effects on disgust, disapproval, anger, and contempt remained significant. Unlike with Democrats, our historicist thinking intervention had no impact on compassion. Hence, our historicist thinking intervention significantly decreased Republicans’ disapproval, anger, contempt, and disgust toward Democrats, suggesting that the reduced animosity of Republicans consists of a decrease in both low- and high-intensity negative emotions.

**Table 2:** Experiment 4: Impact of the historicist thinking intervention on moral emotional responses.

	*M*_Control_ (*SE*)	*M*_*Intervention*_ (*SE*)	*f-*value	Partial η^2^	*p-* value	Rank	Critical *p-*value	Comparison
Disgust	2.67 (.09)	2.28 (.10)	8.25	.02	.002	1	.007	Significant
Disapproval	3.89 (.09)	3.57 (.09)	7.24	.02	.004	2	.01	Significant
Anger	2.71 (.08)	2.41 (.08)	6.68	.02	.005	3	.02	Significant
Contempt	2.28 (.06)	2.11 (.06)	3.89	.01	.025	4	.03	Significant
Disappointment	3.69 (.09)	3.50 (.09)	2.16	.01	.07	5	.04	Non-significant
Hatred	2.14 (.08)	2.00 (.08)	1.74	.004	.09	6	.04	Non-significant
Compassion	2.20 (.07)	2.22 (.07)	.04	.00	.42	7	.05	Non-significant

*Note*. *df* = 395

**Fig 4:**
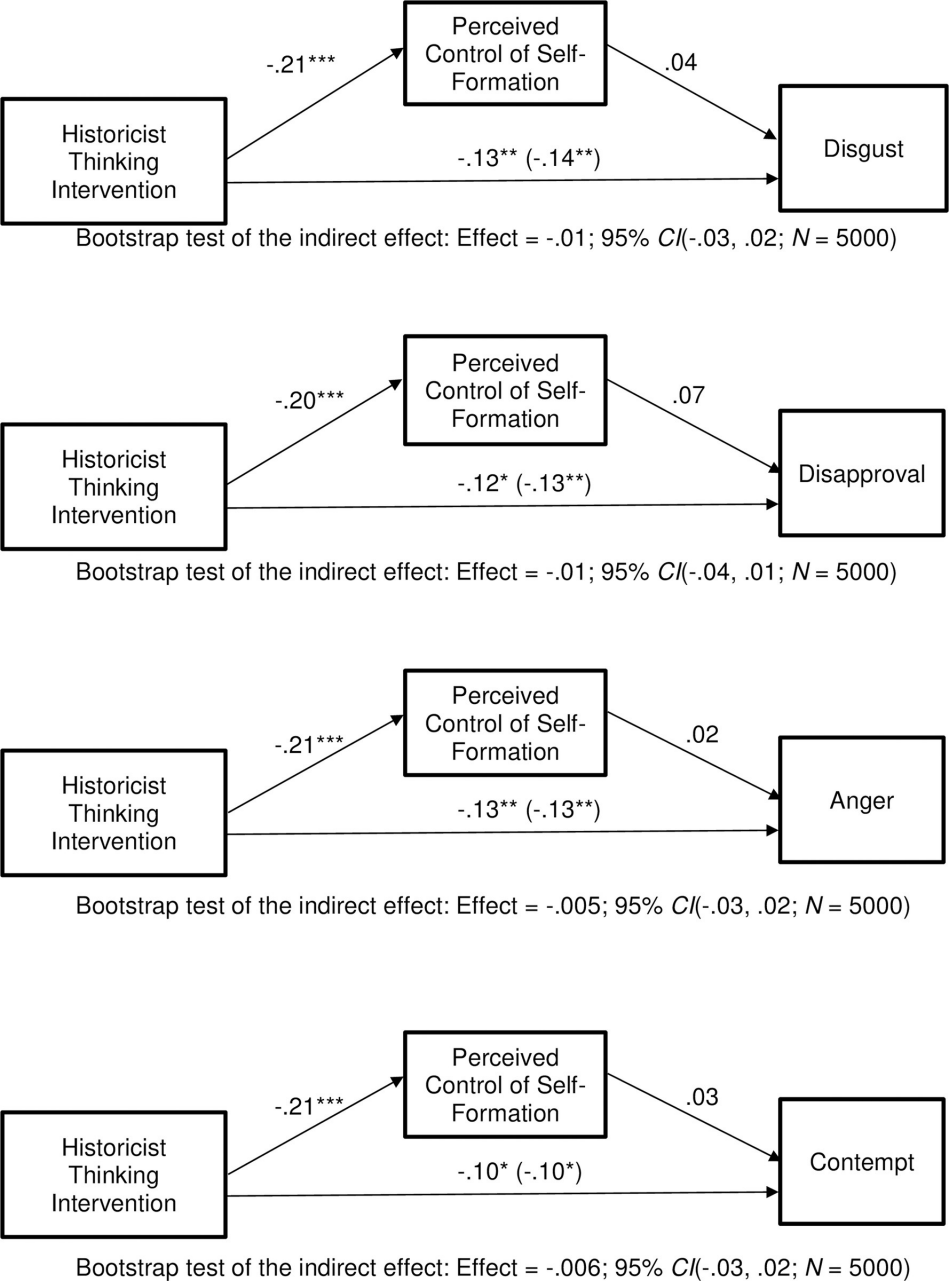
Experiment 4: Among Republicans, the historicist thinking intervention reduces disapproval, anger, contempt, and disgust toward the outparty, but not through perceived control of self-formation. Coefficients are standardized. *p < .05, **p < .01, ***p < .001.

Next, we computed our mediation model. We computed it for disapproval, anger, contempt, and disgust, the four statistically significant effects discussed in the prior paragraph. We used Model 4 from Hayes’ (2018) PROCESS software. All variables were standardized prior to computing the model, so the presented coefficients are standardized beta weights. Results can be seen in Fig 4. As can be seen there, the historicist intervention significantly reduced perceived control of self-formation: *t*(399) = -4.24, *p* < .001 (in the models involving disgust and anger); *t*(398) = -4.17, p < .001 (in the model involving disapproval); *t*(398) = -4.16, p < .001 (in the model involving contempt): effects are slightly different across the models due to missing data. The effects of the intervention on control of self-formation were roughly five times larger than in Experiment 2, possibly due to the revised control of self-formation items. However, perceived control of self-formation was not associated with disgust, *t*(398) = .69, *p* = .25, disapproval, *t*(397) = 1.38, *p* = .08, anger, *t*(398) = .47, *p* = .32, or contempt, *t*(397) = .59, *p* = .28. These were not significant indirect effects, as can be seen by looking at the bootstrap test results beneath the models. The direct effect of our intervention was significant after controlling for the mediator, *t*(398) = -2.66 *p* = .004. Thus, although our intervention reduces animosity toward Democrats among Republicans, this change is not mediated by changes in control of self-formation because perceived control of self-formation, while reduced by our intervention, is unrelated to emotions among Republicans.

Experiment 4, then, provides a conceptual replication of Experiment 2: Our historicist thinking intervention reduces partisan animosity and—for Republicans—this appears to involve reduced feelings of disgust, disapproval, anger, and contempt toward Democrats. These changes in emotions were not, however, mediated by reduced perceptions of control of self-formation (which was also found in Experiment 2, which also focused on Republicans).

## General discussion

Partisan animosity is on the rise in America and is associated with a host of negative outcomes for our democracy [2, 4–7]. Our goal in this article has been to examine a relatively novel intervention to reduce such animosity. We draw on prior work regarding the psychology of blame, specifically, on the role of historicist thinking in mitigating blame [9, 23, 26–31]. Prior work suggests that historicist thinking mitigates blame by generating an understanding that moral violators, in this case, political outparty members, lack control of self-formation and therefore merit reduced blame for who they are.

Experiments 1 and 2 had Democratic and Republican participants, respectively, rate how warm or cold they felt towards the political outparty members as a whole (i.e., using a Feeling Thermometer). Before making this rating, some participants received a historicist thinking intervention. The intervention consisted of three parts: A detailed historicist narrative regarding a specific outparty member, a follow-up “generalizing statement” which suggested that all outparty members have an idiosyncratic story regarding formative influences on their political beliefs, and lastly, an “implications statement" which expressed that outparty members can be changed by future formative experiences just as they have been influenced by past formative experiences. Other participants went directly to making ratings on the feeling thermometer. Results showed that, for both Democrats and Republicans, the historicist thinking intervention reduced cold feelings towards political outparty members as a whole. For Democrats, some of this reduction in animosity was mediated by reduced perceptions of Republican control of self-formation. For Republicans, there was no evidence of mediation via control of self-formation.

We conducted Experiments 3 and 4 for the purpose of attaining a more detailed understanding of what the historicist thinking intervention was changing. We did this by measuring a variety of moral emotions (all of which tap into partisan animosity), as opposed to the global feeling thermometer. Experiments 3 and 4 conceptually replicated Experiments 1 and 2 and also revealed novel information suggesting that our intervention impacted different animosity-relevant emotions in Democrats versus Republicans. Experiments 3 and 4 had Democratic and Republican participants, respectively, rate how strongly they felt compassion, disapproval, disappointment, anger, disgust, contempt, and hatred towards the political outparty. Before making these ratings, some participants received the same historicist thinking intervention employed in the previous experiments. Other participants went directly to making their ratings of moral emotions. Results showed that, for Democrats, the historicist thinking intervention increased compassion toward the political outparty. Like Experiment 1, these changes were partially mediated by reduced perceptions of control of self-formation. For Republicans, the historicist thinking intervention reduced feelings of disapproval, anger, disgust, and contempt toward the political outparty, and like Experiment 2, these changes were not mediated by reduced perceptions of control of self-formation.

Notably, our studies were designed within an “interventionist” framework. Such a framework typically involves creating interventions that are multi-faceted, such as our intervention above which combined a particular historicist story about an individual, a general statement about the role of personal history in shaping each individual, and another general statement about future malleability. One question not answered by our studies, then, is which of these components is most impactful in terms of reducing animosity. Or, might it be possible that they must all be presented together to be effective? Future work could decompose our intervention to examine this issue. Furthermore, future work is also needed to explore mediation of the historicist narrative intervention among Republicans. The intervention clearly works for Republicans in terms of reducing their animosity toward Democrats, but this effect was never mediated by control of self-formation. Is the effect perhaps mediated via increases in perceived malleability? Unfortunately, between the present studies and the studies of Gill et al. [9] we utilized almost all the Republican participants on Prolific! And, naturally, the validity of results will be suspect if the same participants are run in similar studies close in time.

The present studies conceptually replicate previous work which has shown that historicist thinking interventions can reduce blame and verbal hostility towards a political outparty member [9]. The current research extends this line of work, showing that a historicist intervention can reduce partisan animosity towards the outparty *as a whole*, not just towards a particular, hostile member. This is important because animosity toward the outparty as a whole—rather than toward a particular individual—is likely to play an important role in political efforts to exclude, harm, and disempower the outparty [4–7].

Many of the previous approaches we have highlighted for reducing partisan animosity have been categorized as intervening on thoughts, specifically through correcting inaccurate metaperceptions and negative beliefs about outparty members [1, 8, 11, 13, 19]. Other approaches mentioned are based in developing empathy and understanding the perspective of outparty members [10, 15, 16, 18]. Our approach is distinct from these theoretical approaches because it is explicitly derived from work on the psychology of blame. Blame is integral to hostile polarization and, indeed, it can be argued that hostile polarization simply *is* blame: That is, one despises the outparty because they are unethical, untrustworthy, unfair, inhumane, unthinking, and so on–one *blames* them because their beliefs and actions are so profoundly morally wrong. Taking this a step further, it is possible to cast the various interventions to reduce partisan animosity—discussed in the Introduction—in terms of blame mitigation.

To see this clearly, it helps to think of blame as being triggered by two key components [21]: (1) Perception of an objectionable belief or action, and (2) perception that the objectionable belief or action was *freely chosen* and *chosen for bad reasons*. Keeping these ideas in mind, one can see that some interventions work by reducing the sense that objectionable beliefs or actions are widespread within the outparty. For example, the Mernyk et al. [13] intervention—which corrected exaggerated beliefs about the outparty’s support for violence—mitigated blame (i.e., reduced animosity) by reducing the perception that many within the outparty hold objectionable beliefs about violence. Relatedly, the Ahler and Sood [8] intervention—which corrected false beliefs about the percentage of various subgroups in the outparty—arguably operated via this same mechanism (e.g., if only 2% of Republicans are wealthy, then one can no longer blame Republicans for being a bunch of selfish rich people who only care about protecting their assets). One can see that other interventions work by reducing the sense that the outparty’s objectionable beliefs or actions were freely chosen and/or chosen for bad reasons. For example, the Stanley et al. [16] and Kubin et al. [10] interventions mitigated blame (i.e., reduced animosity) by showing that outparty members had compelling arguments for their beliefs (rather than being unthinking or driven by unreasonable or unethical reasons). The Santos et al. [15] intervention—which encouraged people to see the value of having empathy for the outparty—mitigated blame (i.e., reduced animosity) by encouraging people to see outparty members as open to gentle persuasion and compromise (rather than as closed-minded and unlikely to be persuaded).

We recommend that future work examine political polarization through the lens of blame. The psychology of blame can contribute to a richer understanding of toxic polarization, which can inform interventions to tackle partisan animosity. For example, toxic polarization seems likely to be driven, in part, by misplaced *collective* blame, or blaming the outparty as a whole for the extreme acts of a minority within the outparty. If this is correct, then one intervention for reducing partisan animosity may be to encourage *particularizing* blame rather than letting it remain collective. That is, it might be possible to nudge partisans in the direction of perceiving certain individuals and groups within the outparty as far more blameworthy than the outparty as a whole. For example, with regard to the January 6^th^ riot, Democrats might be nudged to perceive Donald Trump and the rioters as highly blameworthy, whereas most Republicans are hardly blameworthy at all. Without a doubt, societies around the world are sorely in need of interventions to temper partisan animosity. We hope that this paper provides a useful contribution to that effort.

## Supporting information

S1 File Supplemental materials. (DOCX)
