# Supplementary material for: Partisan animosity through the lens of blame: Partisan animosity can be reduced by a historicist thinking intervention
Source: PLoS One. 2024 Jan 10;19(1):e0295513. doi: 10.1371/journal.pone.0295513 (PMC10781133; doi:10.1371/journal.pone.0295513)
Supplement: S1 File — (DOCX) [file pone.0295513.s001.docx]

**Supplemental Materials**

**Manipulations and Dependent Variables**

**Historicist Thinking Intervention Text**

**EXPERIMENTS 1 and 3**

**Introduction**

*First, we will have you read about a Republican and how she developed her political beliefs under the influence of family and community.*

**Historicist Narrative**

*This is Sabrina.*

*Sabrina is 19 years old. She grew up in an Evangelical Christian home. Everyone in Sabrina's predominately White, small town rural area also goes to the same Evangelical church and holds the same beliefs as Sabrina and her family. For her entire life, Sabrina has heard from respected leaders of her church that abortion is one of the greatest moral violations in modern times. She has never heard an alternative point of view on the issue. Sabrina’s father works in a manufacturing job and tells Sabrina that conservative trade policies will save his job from going overseas. Neither Sabrina nor her parents went to college. Thus, Sabrina has had very little exposure to outside perspectives, and her family mainly gets information from news sources which consistently argue that conservative values will improve the nation. Sabrina is surrounded by people who constantly speak negatively about liberal values. Sabrina views liberal policies as dangerous and harmful to her family and against her and her family’s traditional values. In short, because Sabrina grew up “in a conservative bubble,” she lacks any real understanding of liberal viewpoints.*

**Generalizability Statement**

*Although Sabrina's story is not the same as the story for all Republicans, it is nevertheless true that every person has a story behind his or her political views. Indeed, every individual's beliefs and attitudes are created in the context of his or her family, personal upbringing, geography, media exposure, spiritual and religious background, educational background, class, etc. No one becomes who they are all by themselves, but rather each person is forged by their surroundings and life experiences.*

**Future Malleability Statement**

*Just as every person's belief system is formed through a variety of formative experiences, belief systems can also change via formative experiences. These formative experiences can include new learning, conversations with those who see things differently, moving to a new part of the country, etc.*

**EXPERIMENTS 2 and 4**

**Introduction**

*First, we will have you read about a Democrat and how she developed her political beliefs under the influence of family and community.*

**Historicist Narrative**

*This is Sabrina.*

*Sabrina is 19 years old. She grew up in a religious community called “The Society of Friends”*

*(commonly known as "Quakers"). The community emphasizes the teachings of Quaker Pacifism,*

*which is the belief that war and violence are against God's wishes. Everyone in Sabrina's tight-*

*knit rural community go to the same Quaker Meeting House and hold the same beliefs as*

*Sabrina and her family. For her entire life, Sabrina has heard from respected leaders of her*

*community that guns and gun ownership are immoral for a civilized society. She has never heard*

*an alternative point of view on the issue. Sabrina’s father tells her that restrictions on gun*

*ownership will stop innocent people from being killed. Neither Sabrina nor her parents went to*

*college. Thus, Sabrina has had very little exposure to outside perspectives, and her family*

*overwhelmingly gets information from news sources that support their beliefs. Sabrina is*

*surrounded by people who constantly speak negatively about conservative values surrounding*

*gun ownership. She views conservative policies as dangerous and in violation of her and her*

*family’s Quaker values. In short, because Sabrina grew up “in a liberal bubble,” she lacks any*

*real understanding of conservative viewpoints on guns*

**Generalizability Statement**

*Although Sabrina's story is not the same as the story for all Democrats, it is nevertheless true that every person has a story behind his or her political views. Indeed, every individual's beliefs and attitudes are created in the context of his or her family, personal upbringing, geography, media exposure, spiritual and religious background, educational background, class, etc. No one becomes who they are all by themselves, but rather each person is forged by their surroundings and life experiences.*

**Future Malleability Statement**

*Just as every person's belief system is formed through a variety of formative experiences, belief systems can also change via formative experiences. These formative experiences can include new learning, conversations with those who see things differently, moving to a new part of the country, etc.*

*.*

**Dependent Variables**

**Control of Self-Formation (Group)**

- [Democrats/Republicans] as a whole have free will in terms of BECOMING people who have [liberal/conservative] views.
- Throughout their lives, [Democrats/Republicans] as a whole are always in control of the development of their political views.
- As a whole, [Democrats’/Republicans’] antagonism toward those with opposing views are purely a result of them freely choosing to become who they currently are.

**EXPERIMENTS 1 and 2**

**Feeling Thermometer**

We would like to get your feelings toward both Democrats and Republicans. We would like you to rate them using something we call the feeling thermometer. Ratings between 50 degrees and 100 degrees mean that you feel favorable and warm toward them. Ratings between 0 degrees and 50 degrees mean that you don't feel favorable toward them and that you don't care too much for them. You would rate them at the 50 degree mark if you don't feel particularly warm or cold toward them.


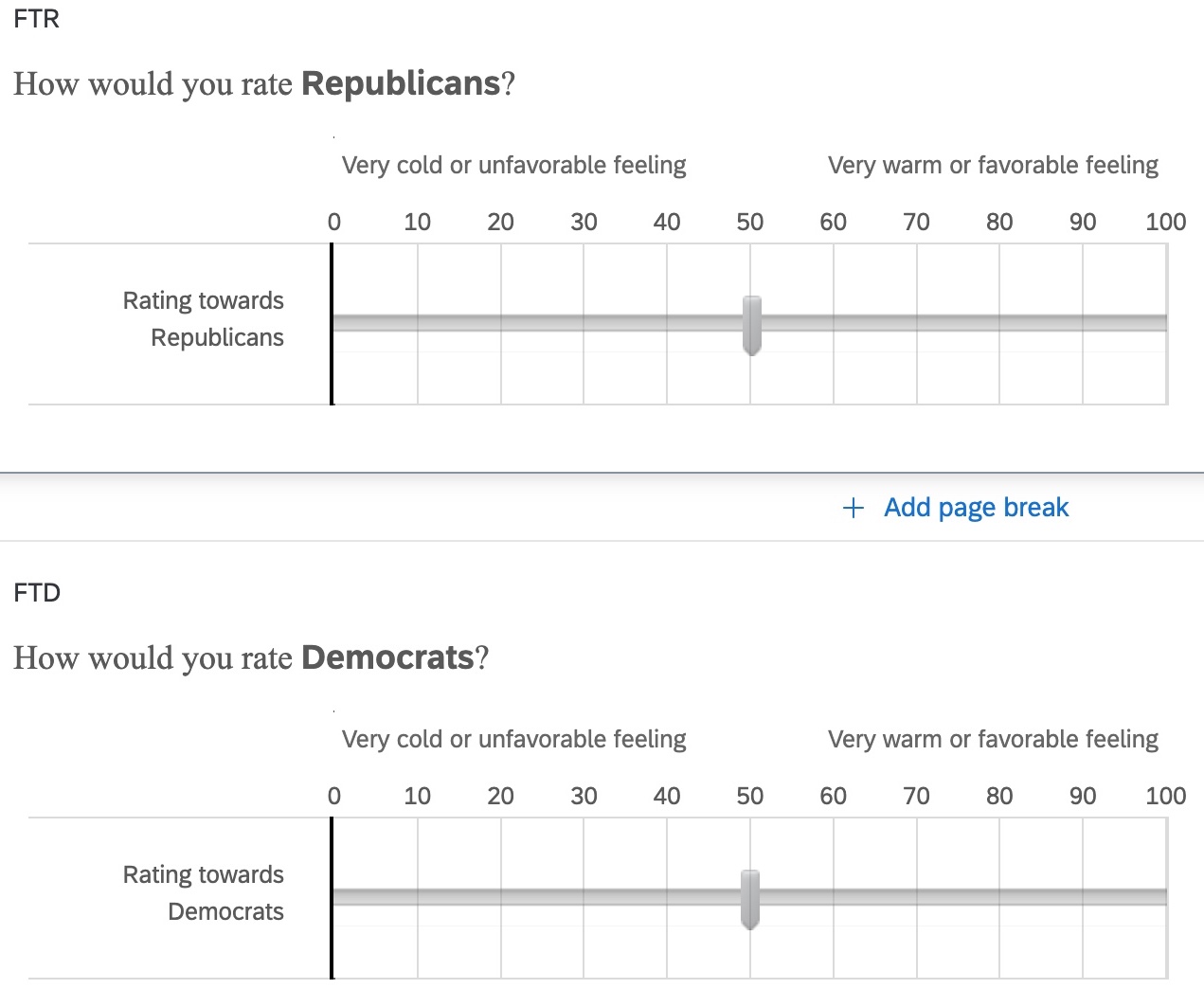


**Dictator Game**

You have been anonymously and randomly matched with another participant who identifies as a Republican. You have just been given 50 cents. You will now decide how to split these 50 cents between yourself and the [Republican/Democrat] participant. You can give any amount between 0 cents and 50 cents to the other participant. The other participant cannot affect the outcome you choose. For example:

-If you give 0 cents, you will end up with 50 cents and the [Republican/Democrat] participant will end up with 0 cents.

-If you give 50 cents, you will end up with 0 cents and the [Republican/Democrat] participant will end up with 50 cents.


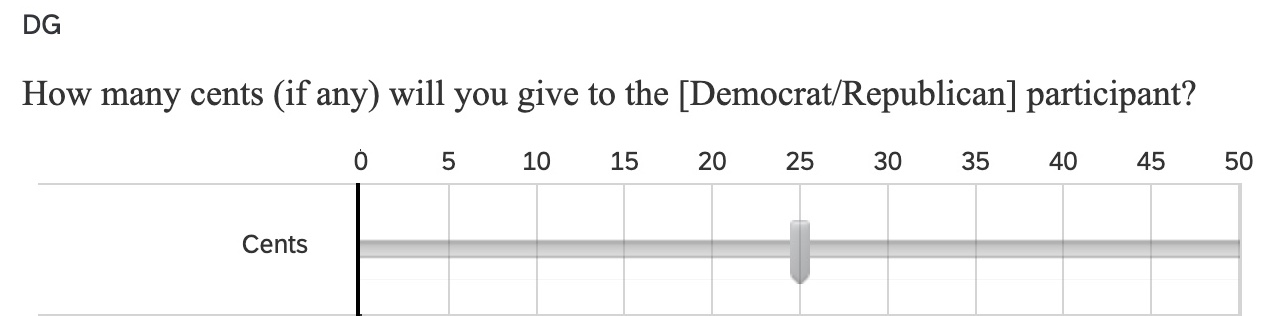


**EXPERIMENTS 3 and 4**

**Emotions**

*Each word was rated on a scale from 1 (not at all) to 5 (strongly).*

Toward [Republicans/Democrats] I feel…

- Anger (3 items)
  - Anger
  - Outrage
  - Infuriated
- Disapproval (1 item)
- Hate (4 items)
  - Hatred
  - Hostility
  - Loathing
  - Scorn
- Compassion (2 items)
  - Compassion
  - Sympathy
- Disgust (3 items)
  - Disgust
  - Repulsed
  - Sickened
- Disappointment (2 items)
  - Disappointment
  - Let down
- Contempt (2 items)
  - Disrespect
  - Contempt

**MISSING DATA**

**EXPERIMENT 1**

Two participants in May did not complete any portion of the survey, and an additional two participants did not complete control of self-formation ratings.

**EXPERIMENT 2**

Two participants from October/November did not complete any portion of the survey.

**EXPERIMENT 3**

One participant did not complete anger, disgust, and hatred ratings, another participant did not complete compassion ratings, five participants did not complete disgust ratings, one participant did not complete disgust and disappointment ratings, two participants did not complete contempt ratings, and one participant did not complete disappointment ratings.

**EXPERIMENT 4**

One participant did not complete disapproval ratings, another participant did not complete compassion ratings, another participant did not complete disappointment ratings, and one participant did not complete contempt ratings.

**ANALYSES NOT PRESENTED IN THE MAIN TEXT**

We examined Democrats and Republicans from Experiments 3 and 4, which were the experiments in which we used our revised control of self-formation measures. Our analysis revealed a significant-yet-small effect of party affiliation, *t*(799) = -2.24, *p* = .025 (*d* = .16; 95% *CI*: -.25, -.02). Democrats had weaker control of self-formation beliefs (*M* = 3.65) compared to Republicans (*M* = 3.78).

**EXPERIMENT 1**

Regarding the *dictator game*, we found no effect of the historicist thinking condition, *t*(1, 699) = -1.48, *p* = .07 (*M_Control_* = 76.23, *M_Intervention_* = 73.46; *d =* -.11, 95% *CI*: -.26, .04).

Regarding the *feeling thermometer,* there was a significant effect of the historicist thinking condition after controlling for Prolific’s collected demographic variables of age, sex, race, and employment status, *F*(1, 500) = 5.06, *p* = .01 (*M_Control_* = 82.64, *M_Intervention_* = 79.01; η^2^ *=* .01, 95% *CI*: .00, .03). None of the control variables were significantly related to feeling thermometer ratings (??).

**EXPERIMENT 2**

Regarding the *dictator game*, we found no effect of the historicist thinking condition, *t*(1, 644) = -1.38, *p* = .08 (*M_Control_* = 69.82, *M_Historicist_* = 67.10; *d = -*.11, 95% *CI*: -.26, .05).

Regarding the *feeling thermometer,* there was a significant effect of the historicist thinking condition after controlling for Prolific’s collected demographic variables of age, sex, race, and employment status, *F*(1, 436) = 3.59, *p* = .03 (*M_Control_* = 70.01, *M_Intervention_* = 66.08; η^2^ *=* .01, 95% *CI*: .00, .03). There was a significant relationship of partisan animosity with age, *F*(1, 436) = 3.94, *p* = .048 (*B =* .16, *SE* = .08, 95% *CI*: .00, .31), suggesting that animosity increased as age increased.

**EXPERIMENT 3**

Regarding the *moral emotions,* there was a significant effect of the historicist thinking condition after controlling for Prolific’s collected demographic variables of age, sex, race, and employment status, *F*(7, 341) = 1.80, *p* = .045, Pillai’s Trace *V* = .04 (η^2^ *=* .04, 95% *CI*: .00, .06). There was a significant relationship of partisan animosity with age *F*(7, 341) = 2.41, *p* = .02. A follow-up test revealed that hatred was the only specific emotion significantly related to age (*B =* -.013, *SE* = .006 95% *CI*: -.02, -.00). There was also a significant relationship of partisan animosity with race *F*(7, 341) = 2.32, *p* = .03, Pillai’s Trace *V* = .05 (η^2^ *=* .05, 95% *CI*: .00, .08). Pairwise comparisons revealed that anger was significantly higher in White Americans (*M* = 3.36) relative to Black Americans (*M* = 2.90), disapproval was significantly higher in White Americans (*M* = 4.28) relative to Black Americans (*M* = 3.83), contempt was significantly higher in White Americans (*M* = 2.59) relative to Asian Americans (*M* = 2.09), and disappointment was significantly higher in White Americans (*M* = 4.10) relative to Asian Americans (*M* = 3.58) and Black Americans (*M* = 3.57).

**EXPERIMENT 4**

Regarding the *moral emotions,* there was a significant effect of the historicist thinking condition after controlling for Prolific’s collected demographic variables of age, sex, race, and employment status, *F*(7, 364) = 2.35, *p* = .01, Pillai’s Trace *V* = .04 (η^2^ *=* .04, 95% *CI*: .00, .07). There was a significant relationship of partisan animosity with age *F*(7, 364) = 4.64, *p* < .001. Follow-up tests revealed that disapproval (*B =* .01, *SE* = .004, 95% *CI*: .00, .02), disappointment (*B =* .02, *SE* = .01, 95% *CI*: .01, .03), and contempt (*B =* .01, *SE* = .003, 95% *CI*: .00, .01) were the specific emotions significantly related to age, with older individuals feeling more of each of these feelings.

**DRAWBACKS OF CORRELATIONAL TESTS OF MEDIATION**

Bullock, J. G., & Green, D. P. (2021). Bullock, J. G., & Green, D. P. (2021). The failings of conventional mediation analysis and a design-based alternative. *Advances in Methods and Practices in Psychological Science, 4*(4), 25152459211047227

Bullock, J. G., Green, D. P., & Ha, S. E. (2010). Yes, but what’s the mechanism?(don’t expect an easy answer). *Journal of personality and social psychology*, *98*(4), 550
